# Mechanistic Insight into PPARγ and Tregs in Atherosclerotic Immune Inflammation

**DOI:** 10.3389/fphar.2021.750078

**Published:** 2021-09-29

**Authors:** Zhao Gao, Xinrui Xu, Yang Li, Kehan Sun, Manfang Yang, Qingyue Zhang, Shuqi Wang, Yiyi Lin, Lixia Lou, Aiming Wu, Weijing Liu, Bo Nie

**Affiliations:** ^1^ Key Laboratory of Chinese Internal Medicine of Ministry of Education and Beijing, Dongzhimen Hospital Affiliated to BeijingUniversity of Chinese Medicine, Beijing, China; ^2^ Zhanjiang Key Laboratory of Prevention and Management of Chronic Kidney Disease, Institute of Nephrology, Guangdong Medical University, Zhanjiang, China; ^3^ School of Traditional Chinese Medicine, Beijing University of Chinese Medicine, Beijing, China

**Keywords:** atherosclerosis, peroxisome proliferator-activated receptor gamma, tregs, inflammation, immunoregulation

## Abstract

Atherosclerosis (AS) is the main pathological cause of acute cardiovascular and cerebrovascular diseases, such as acute myocardial infarction and cerebral apoplexy. As an immune-mediated inflammatory disease, the pathogenesis of AS involves endothelial cell dysfunction, lipid accumulation, foam cell formation, vascular smooth muscle cell (VSMC) migration, and inflammatory factor infiltration. The nuclear receptor peroxisome proliferator-activated receptor gamma (PPARγ) plays an important role in lipid metabolism, inflammation, and apoptosis by antagonizing the Wnt/β-catenin pathway and regulating cholesterol efflux and inflammatory factors. Importantly, PPARγ-dependant fatty acid uptake is critical for metabolic programming. Activated PPARγ can exert an anti-atherosclerotic effect by inhibiting the expression of various inflammatory factors, improving endothelial cell function, and restraining the proliferation and migration of VSMCs. Regulatory T cells (Tregs) are the only subset of T lymphocytes that have a completely negative regulatory effect on the autoimmune response. They play a critical role in suppressing excessive immune responses and inflammatory reactions and widely affect AS-associated foam cell formation, plaque rupture, and other processes. Recent studies have shown that PPARγ activation promotes the recruitment of Tregs to reduce inflammation, thereby exerting its anti-atherosclerotic effect. In this review, we provide an overview of the anti-AS roles of PPARγ and Tregs by discussing their pathological mechanisms from the perspective of AS and immune-mediated inflammation, with a focus on basic research and clinical trials of their efficacies alone or in combination in inhibiting atherosclerotic inflammation. Additionally, we explore new ideas for AS treatment and plaque stabilization and establish a foundation for the development of natural PPARγ agonists with Treg recruitment capability.

## Introduction

Atherosclerosis (AS) is the main pathological basis of coronary AS, heart disease, cerebral infarction, and peripheral vascular diseases, which seriously threatens health and even lives (Scannapieco et al., 2003). The etiology and pathogenesis of AS are complex and involve various pathological processes from the onset of AS to plaque formation, rupture, and thrombosis, although, the specific mechanisms have not been fully clarified. Studies have implicated inflammation through the entire process of AS, and various cytokines and inflammatory factors act on different stages of AS (Ross, 1999).

Peroxisome proliferator activated receptor γ (PPARγ) is a type of nuclear transcription factor activated by ligands. PPARγ can regulate lipid metabolism, improve insulin resistance, suppress the transformation of macrophages into foam cells and their deposition on the blood vessel wall (Plutzky, 2000; Hsueh and Law, 2001). Furthermore, PPARγ agonists can antagonize the activation of the WNT/β-catenin pathway and affect metabolic reprogramming, attenuate the glycolytic pathway, repress excessive lactate production, and reduce mitochondrial damage, thereby inhibiting inflammation to exert its anti-atherosclerotic effect(Angela et al., 2016; Vallée and Lecarpentier, 2018).

Regulatory T cells (Tregs) are a special subset of T cells discovered in recent years. They play a crucial role in the regulation of immune tolerance, termination of the activated immune response, maintenance of immune homeostasis, and regulation of effector T lymphocytes (Veeken et al., 2016). Tregs have been implicated in delaying the progression of AS, mainly by inhibiting the secretion of cytokines, producing immunosuppressive enzymes, inhibiting macrophage inflammation, reducing plaque vulnerability, directly killing effector T cells, and regulating the maturation and function of dendritic cells (DCs) (Taleb et al., 2008; Ou et al., 2018).

Both PPARγ and Tregs are involved in immune regulation. PPARγ participates in the regulation of inflammation and the immune response, and is closely related to immune inflammation. Recent studies suggest that metabolism affects immune inflammation, and metabolic reprogramming plays an importance role in the regulation of immune inflammation, which is currently a hot topic of research (Ganeshan and Chawla, 2014). Tregs, as the main immune negative regulator cells, play a vital role in maintaining immune balance, inhibiting inflammatory response. Further, the activation of PPARγ can recruit Tregs, which in turn can inhibit the inflammatory response leading to significant anti-AS effects (Cipolletta et al., 2012; Hamaguchi and Sakaguchi, 2012). Given the role of PPARγ and Tregs in AS, this review focuses on the anti-inflammatory regulatory role of PPARγ and Tregs in the progression of AS and the mechanisms, involved, in order to provide a theoretical basis for further exploring the relationship between PPARγ and Tregs, and to promote novel approaches for the treatment of AS and stabilization of AS plaques.

### The Complex Pathological Processes of Atherosclerosis

AS refers to the disease of medium and large arteries, which commonly manifests as endothelial dysfunction, endometrial lipid deposition, smooth muscle cell proliferation, cell apoptosis, and necrosis, as well as systemic and local inflammation.(Libby, 2021). AS pathogenesis is a complex process and many hypotheses have been formulated in an attempt to understand its underlying mechanisms, such as the lipid infiltrative theory (Dargel, 1989), oxidative stress theory (Harrison et al., 2003), and vascular smooth muscle cell (VSMC) cloning theory (Suttles et al., 1995). However, no single hypothesis can fully explain the pathological mechanisms of AS. In 1999, Ross proposed the concept of “AS is a chronic inflammatory disease”, revealing that AS involves a process of vascular damage caused by the interaction between vascular wall cells and blood cells combined with inflammatory and proliferative factors (Ross, 1999). These changes reveal that the immune inflammatory response is the first step in the formation of the AS plaque, and is also an important reason for the instability of atherosclerotic plaque. During the pathological process of AS, the increase in phagocytosis of ox-LDL by macrophages, leads to the formation of foam cells and their accumulation to form “Fatty Deposits” on the vascular wall, which is first macroscopic sign of atherosclerotic disease(Libby et al., 2019; Ali et al., 2020; Khatana et al., 2020; Munjal and Khandia, 2020). As the disease progresses, lipid-rich plaques develop. (Alexander and Owens, 2012; Cornelissen et al., 2019). Smooth muscle cells (SMC) migrate into the intima and prolificate, gradually forming the fibrous cap of the plaque, which is a critical event in the progression of AS lesions (Bennett et al., 2016). Meanwhile, under the stimulation of inflammation, activated endothelial cells, macrophages, and foam cells release pro-inflammatory cytokines and proteolytic enzymes, resulting in the thinning of plaque fibrous caps, the gradual instability of plaque, the formation of vulnerable plaques, and the central link of plaque rupture (Sevuk et al., 2015; Kolodgie et al., 2017). Therefore, taking measures to preventing of AS inflammatory response and increasing plaque stability have become an important area of research.

## Atherosclerosis and the Immuno-Inflammatory Response

### Atherosclerosis and Inflammation

Inflammation is the body’s defense response to irritants and is an important part of the immune system’s ability to perform its functions and maintain the body’s homeostasis (Hansson and Hermansson, 2011; Geovanini and Libby, 2018). In 1986, Ross first proposed AS as an inflammatory disease (Ross, 1986), and in 1999, explicitly emphasized that AS is a chronic progressive inflammatory disease (Ross, 1999). Over the past 20 years, extensive research has been carried out investigating AS inflammation. Maintaining the integrity of endothelial cells, inhibiting the migration and proliferation of smooth muscle cells, and preventing plaque rupture can effectively delay the progression of AS by inhibiting inflammation (Zhu et al., 2018; Bäck et al., 2019; Simonetto et al., 2019; Wolf and Ley, 2019; Das and Natarajan, 2020). Thus, the inflammatory response plays an important role in the initiation and development of AS.

### Atherosclerosis and Immunity

Immunity refers to a physiological protective function of the body, including a series of processes such as recognition, elimination, and destruction of foreign substances, pathogenic organisms or non-altering organisms. Excessive or insufficient immunity can cause damage to tissues (Wolf and Ley, 2019). The immune response is present throughout the development of AS. The human immune response is divided into two main parts: innate immunity and adaptive immunity. Innate immunity is a natural defense system that is available from birth. The components involved in the immune system mainly include phagocytic cells, natural killer (NK) cells, lysozymes, and the complement, which play a non-specific defense role against foreign antigens. Adaptive immunity is an immune response that occurs when the body responds to a specific antigen. Antigen-presenting cells (APC) (DCs, macrophages) recognize and process antigens and present them to T or B lymphocyte surface receptors to induce a series of specific immune responses. A variety of immune cells, including monocyte-macrophages and T cells, involved in adaptive and innate immunity are active in the pathogenesis of AS. These immune cells regulate the balance between pro-AS inflammation and anti-AS inflammation through complex interactions, and participate in promoting/inhibiting AS lesions or reducing/increasing the stability of the AS plaque (Schaftenaar et al., 2016; Kobiyama and Ley, 2018; Bartoloni et al., 2019; Herrero-Fernandez et al., 2019; Zhao and Mallat, 2019).

#### Atherosclerosis and Innate Immunity

The innate immune cells mainly include mononuclear-macrophages and DCs. Macrophages are most closely associated with the occurrence and development of AS. In response to inflammatory signals, AS-associated immune cells initiate a process of “metabolic reprogramming”, defined by changes in cellular metabolic energy supply pathways (Ganeshan and Chawla, 2014) with a shift from oxidative phosphorylation (OXPHOS) to aerobic glycolysis, which is related to glucose uptake and glycolysis(Rashida Gnanaprakasam et al., 2018). Glycolysis is the main process through which macrophages obtain energy, and metabolic reprogramming is essential for maintaining macrophage activation and functions. Physiologically, human monocytes and macrophages are derived from the bone marrow, and enter the blood and tissues after differentiation and maturation. In the pathological state of AS, monocytes in the blood cross the endothelium gap into the subendothelial layer, and are transformed into macrophages under the stimulation of chemokines. The scavenger receptors modify lipoproteins by recognizing the proteases and oxygen free radicals they produce, to accelerate the ingestion of oxidatively modified low-density lipoprotein (ox-LDL) by macrophages, and which in turn transform into foam cells (Wang et al., 2019). In addition, metabolic conversion from fatty acid oxidation (FAO) and OXPHOS to glycolysis was also found in ox-LDL-stimulated pro-inflammatory macrophages (Sun et al., 2020). Excessive accumulation of foam cells forms atheromatous plaques. Macrophages can be polarized into M1 and M2 phenotypes in different microenvironments. Furthermore, M1 type macrophages are mainly induced by interferon (IFN) γ and lipopolysaccharide (LPS), and exert strong phagocytic activity, and secrete tumor necrosis factor (TNF)-α and other pro-inflammatory factors, inducing T helper 1(Th1) type cellular immune responses that mediate inflammation (Verreck et al., 2004; Vogel et al., 2013). M2 type macrophages are induced by interleukin (IL)-13 and can secrete many anti-inflammatory cytokines such as IL-10, to attenuate inflammation and promote tissue damage repair (Shirey et al., 2008; Martinez et al., 2009). Previous studies have shown that M1-type macrophages can induce smooth muscle cell proliferation and the release of vasoactive molecules such as nitroxide (NO) and endothelin, to promote the oxidation of LDL and induce cytotoxicity. Early AS plaques can be infiltrated by M2 type macrophages, but with disease progression, the number of M1 type macrophages increases and becomes dominant (Kirbiš et al., 2010). The increase of M1 type macrophages will promote the occurrence and development of AS, and together with transforming growth factor-β (TGF-β) released by M2-type macrophages can inhibit the recruitment of inflammatory cells, thus slowing down the development of AS (Shapouri-Moghaddam et al., 2018).

#### Atherosclerosis and Adaptive Immunity

Adaptive immune cells include T cells and B cells. T cells are closely associated with the development of AS. They exert pro-inflammatory or anti-inflammatory effects by secreting different cytokines or antibodies, and can also change phenotype according to the microenvironment, which suggests that T cells may have plasticity. In the process of AS, effector T cells secrete corresponding cytokines to induce differentiation into other cell subtypes (Munjal and Khandia, 2020). T cells can be divided into two major subgroups according to the Cluster of Differentiation (CD), CD4^+^ T cells and CD8^+^ T cells (Gebhardt et al., 2011). Among these, naïve CD4^+^ T cells are the most closely associated with AS (Arranz et al., 2015; Grönberg et al., 2017). CD4^+^ T cells can differentiate into different T helper cell subsets such as T helper 1 (Th1), T helper 2 (Th2), T helper 17 (Th17), and Tregs according to the different environments present *in vivo* (Gao et al., 2019; Saigusa et al., 2020). Th1 cells promote the development of AS by secreting IFN-γ, TNF-α, and IL-2 (Baidya and Zeng, 2005). Th2 cells regulate the progression of AS inflammation by secreting anti-inflammatory factor IL-13 and pro-inflammatory IL-4. IL-13 is active against AS by stimulating macrophages to polarize into the M2 subtype and by releasing IL-10 and TGF- β, as well as by activating the signal transducer and activator of transcription 3 (STAT3) (Cardilo-Reis et al., 2012; Brunner et al., 2017; Bi et al., 2020). However, IL-4 can increase the expression of CD36 on macrophages to intensify the phagocytosis of macrophages, and promote the progress of AS (Lee and Hirani, 2006). By secreting IL-1β, IL-6, IL-17, TNF-α, and other pro-inflammatory factors. Th17 accelerates the progression of AS (Taleb et al., 2015; Allam et al., 2018; Munjal and Khandia, 2020). Tregs suppress the immune response and inflammatory reaction by secreting TGF-β and IL-10 to exert anti-AS effects (Pastrana et al., 2012). Thus, the above evidence indicates inflammation is an important pathological factor causing AS, and the regulation of AS inflammation depends on immune cells, inflammatory factors, and other mediators. In recent years, studies on inflammatory immune response associated with AS have provided new approached for the treatment of AS.

## Nuclear Receptor Peroxisome Proliferator-Activated Receptor Gamma and as Inflammatory Response

**FIGURE 1:**
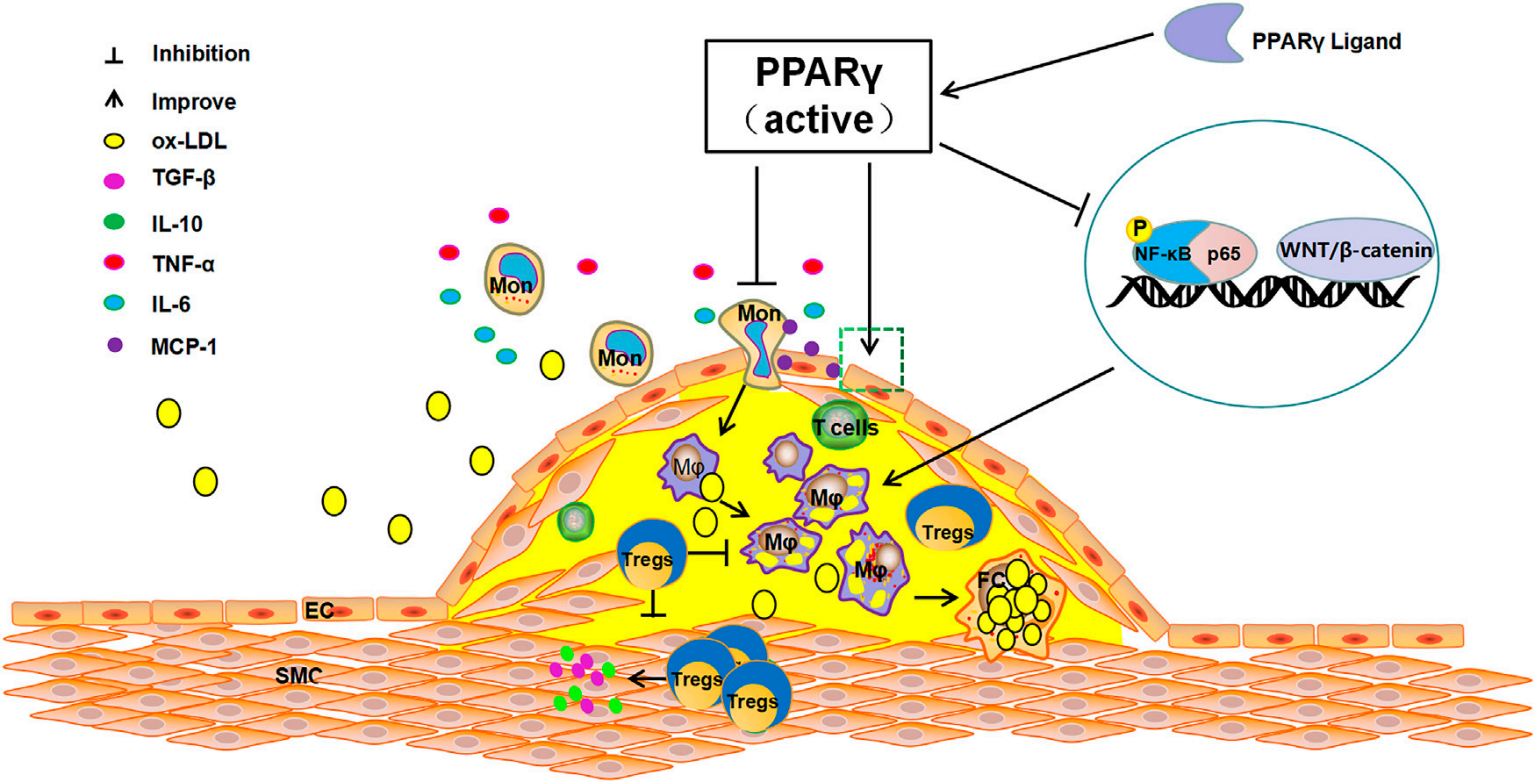
The anti-atherosclerosis pathways of PPARγ. The activation of PPARγ can recruit Tregs to improve endothelial function, inhibit the release of TNF-α, IL-6, and other inflammatory cytokines, reduce the production of MCP-1, suppress the differentiation of monocytes to macrophages and foam cells. In contrast, PPARγ activation can directly inhibit NF-KB phosphorylation or can indirectly inhibit the activation of NF-KB by competitively binding p65 to reduce the production of pro-inflammatory cytokines and restrain AS.

Many cytokines are involved in the progression of AS, among which the nuclear receptor PPARγ has become an important regulatory factor in the inflammatory response (Ivanova et al., 2015). PPARγ is a member of the nuclear receptor transcription factor superfamily that regulates the expression of target genes. It is mainly expressed in adipose tissue and the immune system, and is closely related to adipocyte differentiation, body immunity, insulin resistance, and vascular inflammation (Harris and Phipps, 2001; Finck et al., 2008; Pastrana et al., 2012). PPARγ has a critical role in lipid metabolism, promoting free fatty acid uptake and triacylglycerol accumulation in adipose tissue and liver (Ahmadian et al., 2013). A large number of studies have shown that activation of PPARγ can inhibit the development of AS and stabilize plaques by suppressing the expression of various inflammatory factors, improving endothelial cells function, restraining the differentiation of monocytes into macrophages and the proliferation and migration of smooth muscle cells (Gao et al., 2011; Sanz et al., 2012). Activating PPARγ can directly affect the anti-inflammatory effects on vascular walls, attenuates the inflammatory response of blood vessels, and reduces the formation of foam cells (McCarthy et al., 2013). PPARγ inhibits the release of inflammatory factors such as TNF-α and IL-6 produced by activated monocytes, decreases the production of monocyte chemotactic proteins and reduces the aggregation of monocytes (Ricote et al., 1999; McCarthy et al., 2013; Lim et al., 2015). Additionally, when AS occurs, the WNT/β-catenin pathway is activated and PPARγ expression is down-regulated. The activation of the WNT/β-catenin pathway enhances transcription of target genes involved in inflammation, endothelial dysfunction, and VSMC proliferation. However, the administration of PPARγ agonists antagonizes the above phenomena and inhibits the WNT/β-catenin pathway, with consequent suppression of the AS inflammatory response (Vallée and Lecarpentier, 2018). In the state of AS inflammation, the key macrophage inflammatory gene expression lies in the activation of Nuclear factor-κB (NF-κB). PPARγ acts as an upstream regulatory switch of the NF-κB pathway to directly inhibit NF-κB phosphorylation. Conversely, PPARγ can competitively bind p65 with NF-κB to indirectly inhibit the activation of NF-κB, which leads to reduced phagocytosis of ox-LDL by macrophages, thus slowing down the process of AS (Olefsky and Glass, 2010; Jongstra-Bilen et al., 2017; Sykes and Bennett, 2018) (Figure 1)**.**Studies have shown that PPARγ is the target of DNA methyltransferase 1 (DNMT1), which is involved in regulating DNA methylation, and DNMT1-mediated repression of PPARγ leads to worsening of AS inflammation (Yu et al., 2016). Thus, activation of PPARγ can effectively prevent and treat the progression of AS via different pathological processes.

Clinical trials have reported that compared with glimepiride, pioglitazone, a PPARγ agonist, significantly improves insulin resistance and inflammatory reactions of the left main coronary artery in diabetic patients, independently of hypoglycemic effects (Nitta et al., 2013). For pre-diabetic patients, PPARγ agonists can significantly attenuate the annual growth rate of carotid intima-media thickness (CIMT).This clinical effect does not depend on the control of other risk factors (including blood glucose, insulin sensitivity index, blood lipid, adiponectin, or plasminogen activator-1) (Saremi et al., 2013). Japanese researchers determined that long-term (2.5–4 years) use of PPARγ agonists significantly reduced carotid intima-media thickness (Yamasaki et al., 2010). The much-profiled PERISCOPE trial proved that treatment with the PPARγ agonist pioglitazone for 18 months, reduced the percentage of atherosclerotic plaque volume (PAV) by 0.16%, while the PAV in glimepiride control group increased by 0.73% base on intravascular ultrasound evaluation (Nissen et al., 2008). Both basic and clinical studies have shown that activated PPARγ plays multiple roles in suppressing AS inflammatory reaction, alleviating/stabilizing plaques, and reducing acute cardiovascular events.

## Regulatory T Cells and Atherosclerosis Inflammatory Response

### Structure, Classification, and Function of Regulatory T Cells

Tregs are a special T cell subgroup that can regulate the functions of various immune cells (Ou et al., 2018). CD4^+^ T cells can be divided into CD4^+^CD25^−^ effector T cells (Teff) and CD4^+^CD25^+^ Tregs according to the expression of the surface molecule CD25 (Liu et al., 2006). Activated Teff promotes the immune response, while Tregs inhibit the immune response (Amin et al., 2017). Tregs can be divided into natural Tregs (nTregs) and inducible Tregs (iTregs) (Haribhai et al., 2016). Tregs can regulate different immune responses, among which the natural CD4^+^CD25^+^Foxp3^+^ Tregs are the most important (Luz-Crawford et al., 2013). Foxp3, a forkhead box transcription factor, is a specific morphological and functional marker of Tregs and plays an important role in Tregs activity (Schmidt et al., 2016).

Tregs main role is to participate in the regulation of the body’s immune tolerance, terminating activated immune responses, maintaining immune homeostasis, and inhibiting Teff (Veeken et al., 2016). Tregs kill inflammatory cells by secreting granzymes (Libby, 2015). Tregs regulate immune balance by producing immunosuppressive cytokines, such as TGF-β and IL-10 (Hang et al., 2019). Tregs can inhibit the activation of Teff by directly inhibiting the expression of CD80 and CD86 on the surface of APCs through a cytotoxic T-lymphocyte-associated antigen-4 (CTLA-4)-dependent mechanism (Wing et al., 2008). In addition, Tregs can inhibit the proliferation and differentiation of effector T cells via contact with other effector T cells or by secreting immunosuppressive factors such as TGF-β and IL-10. The relationship between the degree of immune inflammatory response and AS has been most widely studied. There is evidence that Tregs inhibit the expression of pro-inflammatory IFN-γ, IL-4, IL-17, IL-6, and chemokines secreted by Th1, Th2, and Th17 in atherosclerotic plaques through the release of TGF-β and IL-10 (Albany et al., 2019; Ait-Oufella et al., 2021), which exert anti-AS effects.

### Mechanism of Regulatory T Cells-Mediated Inhibition of Atherosclerosis Inflammation

**FIGURE 2:**
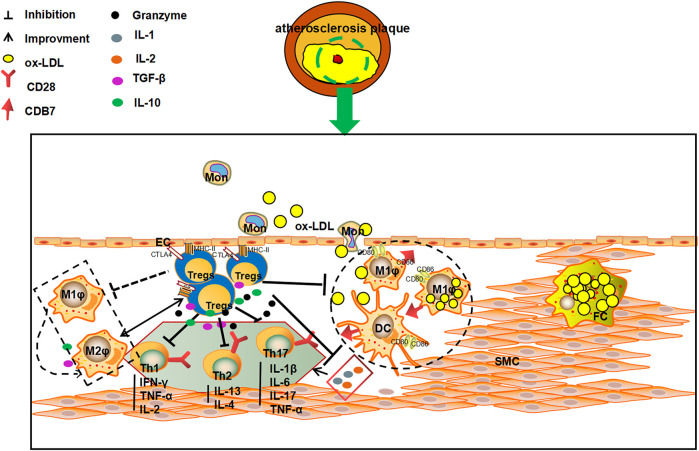
The multiple anti-AS mechanisms of Tregs. Tregs can kill effector T cells via direct contact with T cells, and inhibit the secretion of pro-inflammatory cytokines and the production of immunosuppressive enzymes by secreting TGF-β and IL-10. Tregs can also 1) inhibit the secretion of IL-1 and IL-2 to influence the proliferation of effector T cells by secreting TGF-β and IL-10; 2)suppress the secretion of IFN-γ, TNF-α and IL-4 by Th1 and Th2 cells; and 3) reduce the expression of MHC II on DC and macrophages membrane, and affect the stimulation of CD28/B7 pathway on T cells, and consequently play an important role in inhibiting, slowing down the process of AS and increasing the stability of plaques.

The level and numbers of Tregs is a key factor in AS. Tregs can participate in the pathological process of AS by intercellular contact by inhibiting cytokine secretion and via immunosuppressive enzyme production. In addition, Tregs can also activate killer effector T Cells, and regulating the maturation and function of DCs (Ait-Oufella et al., 2006; Mallat et al., 2007; Chistiakov et al., 2015). Intercellular contact is the main pathway through which Tregs exert their immunomodulatory role. Tregs can complex with major histocompatibility complex-II (MHC- II) through the T cell receptor (TCR) and CTLA-4 to stimulate CD80/CD86 molecule expression on APCs, hence, intercellular contact and negative immune regulation serve as the primary defense system against AS (Sansom, 2000). *In vitro* experiments have shown that deletion of CD86 and CD80 genes significantly reduce the expression of Tregs in a mouse model, as a result, the AS plaque lesion area is amplified (Pastrana et al., 2012). Furthermore, Tregs also suppress the inflammatory response and stabilize AS plaques. Tregs also modulate the secreting of TGF-β and IL-10 and help in the prevention of immune disorders. TGF-β can inhibit the secretion of IL-1 and IL-2, and thus, affect the proliferation of Teff cells. IL-2, which is secreted by Th1 and Th2 cells, can be inhibited by IL-10. IL-10 can also act as a suppresser of DCs and macrophages expressing MHC-II. At the same time, inhibition of the CD28/B7 pathway leads stimulation of T cell proliferation and activation. Thereby, the inflammation response and AS progression can be effectively attenuated and the plaque stability augmented (Chistiakov et al., 2015). Tregs can promote the metabolism of tryptophan through CTLA-4, reduce levels of tryptophan and inhibit Teff (Sojka et al., 2009). Lastly, Tregs can also induce immune cells apoptosis and suppress inflammation of AS immune cells by via perforin or granase (Vignali et al., 2008). (Figure 2)**.**

ApoE^-/-^ mice treated with a high-fat diet showed a decreased number of Tregs and an increased incidence of AS, however treatment with Tregs may prevent the formation of AS plaques (Wang et al., 2014). Tregs can inhibit the formation of foam cells *in vitro* and induce the differentiation of macrophages towards the M2 phenotype (anti-inflammatory phenotype), and the protective cytokine IL-10 secreted by Tregs can prevent the formation of fatty streaks and atherosclerotic plaques (Liu et al., 2011). Zhang et al. showed that in the AS-vulnerable plaque model, Tregs sorted and purified *in vitro* can stabilize vulnerable plaques and reduce the rupture rate of plaques by inhibiting the expression of MMP-2 and MMP-9 and other cytokines in a dose-dependent manner, via TGF-β and IL-10 secretion by Tregs (Meng et al., 2013). Kasper et al.’s *in vivo* studies confirmed that Tregs deficiency or inactivation disrupted the stability of the body’s immune internal environment, and leads to the amplification of the inflammatory response and worsened the progression of AS (Kasper et al., 2016). The size of the AS plaque increased significantly in CD4^+^CD25^+^Treg-deficient mice. Transferring the wild-type (WT) Tregs of mice to ApoE^-/-^ mice, the plaques of the aortic sinus were found to be significantly reduced (Mor et al., 2007). The area of AS plaque increased in Foxp3^+^Tregs deficient mice (Salomon et al., 2006). These studies suggested that by increasing the number of Tregs and enhancing functions, AS can be effectively prevented and treated, findings that are promising for transferring basic research to clinical application.

Clinical studies have shown that Tregs are closely associated with the occurrence of acute coronary syndrome (ACS) and AS plaque vulnerability, while the development of ACS is associated with low levels of circulating immunosuppressive Tregs (Dietel et al., 2013) and increased infiltration of pro-inflammatory DC and Teff cells (Rohm et al., 2015). The levels of Tregs and IL-10 in unstable patients were significantly lower than those in stable patients (George et al., 2012). Few reports have evaluated the effects of drugs on Tregs in AS. Statins can also significantly improve the number and function of Tregs in the peripheral blood of healthy individuals and of AS patients (Rodríguez-Perea et al., 2015). Based on the above animal studies and clinical evidence, a strategy of targeted regulation of Tregs is expected to become an important immunotherapy for the prevention and treatment of cardiovascular and cerebrovascular diseases caused by AS.

## Peroxisome Proliferator-Activated Receptor Gamma, Regulatory T Cells, and Atherosclerosis Inflammation

### Peroxisome Proliferator-Activated Receptor Gamma and Regulatory T Cells

In 2012, a study published in Nature found that activated PPARγ is a key molecule that regulates the accumulation, phenotype, and function of Tregs, and inhibits visceral adipose tissue (VAT) inflammation. PPAR-γ expression by VAT Treg cells was necessary for the complete restoration of insulin sensitivity in obese mice by the thiazolidinedione drug pioglitazone (Cipolletta et al., 2012). Studies have shown that PPARγ can mediate the metabolic reprogramming of macrophages by mediating fatty acid and cholesterol synthesis, and can modify the polarization of macrophages toward anti-inflammatory or pro-inflammatory phenotypes (M2 or M1) to regulate the immune system. There are significant differences in the metabolic reprogramming of macrophages with different phenotypes. For instance, M1 macrophages obtain energy through the anaerobic glycolysis pathway; M2 macrophages produce adenosine triphosphate (ATP) through aerobic glycolysis pathway (Rodríguez-Prados et al., 2010). Interference with cell metabolism by Tregs expressing PPARγ could inhibit adipose tissue inflammation in obese patients, and thus, Tregs may become a new target for the prevention and treatment of inflammation and insulin resistance (Hamaguchi and Sakaguchi, 2012).

Treg aggregation relies on PPARγ pathways in many organs and tissues. Oral polyunsaturated fatty acids (n-3 PUFA) can promote the proliferation of liver Tregs and prevent Con A-induced liver injury, which is achieved through the upregulation of PPARγ and TGF-β by free fatty acids (Lian et al., 2015). By increasing the percentage of CD4^+^CD25^+^Foxp3^+^ Tregs and decreasing the percentage of CD3^+^CD4^+^IFNγ^+^T cells and CD3^+^CD4^+^IL-4^+^ T cells, pioglitazone can alleviate the liver and spleen injury of mice infected with *Schistosoma japonicum*. The mechanism of action is achieved by upregulating the expression of PPARγ and Foxp3 (Zhu et al., 2018). In bronchiolitis obliterans caused by lung transplantation, treatment with the PPARγ agonist pioglitazone can significantly increase the Treg-specific marker Foxp3 and can decrease the expression of inflammatory markers, such as TNF-α, IL-2, so as to reduce lung transplantation postoperative inflammation (Neujahr et al., 2009). In the stomach, *Helicobacter pylori* can reduce insulin resistance through a PPARγ-dependent mechanism and modulates macrophage and Treg infiltration into the abdominal white adipose tissue (Bassaganya-Riera et al., 2012). The transcriptional regulators BATF and IRF4 were necessary for VAT-Treg differentiation through direct regulation of ST2 and PPARγ expression (Vasanthakumar et al., 2015). These effects and mechanisms of Tregs are also realized by PPARγ activation.

### Mechanism of Peroxisome Proliferator-Activated Receptor Gamma- and Regulatory T Cells Mediated Inhibition of Atherosclerosis Inflammation

**FIGURE 3:**
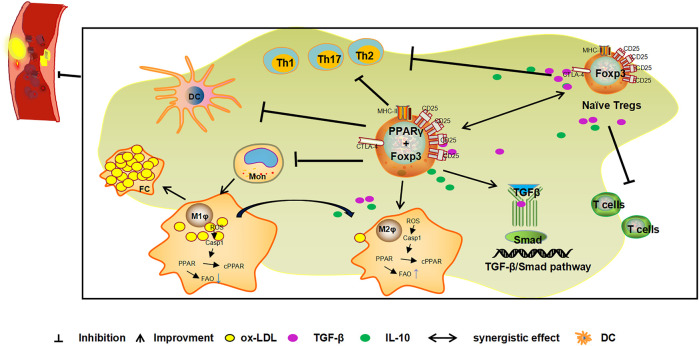
Mechanism of the combined action of PPARγ and Tregs against AS. Tregs expressing PPARγ in VAT exert inhibitory effects on immune cells activity and prevents the occurrence and development of inflammation. Foxp3 is a specific molecule of Tregs. The ectopical co-expression of Foxp3 and PPARγ can induce an increase in Treg levels and the release of cytokines such as TGF-β and IL-10 to inhibit inflammation and exert anti-AS effects. In addition, PPARγ indirectly induces the expression of Tregs by promoting the transformation of macrophages to the anti-inflammatory M2 type and induces the secretion of cytokines such as TGF-β and IL-10 by M2 type macrophages. PPARγ also inhibits the inflammatory response and plays a role in the anti-AS by activating the TGF-β/Smad signalling pathway and inhibiting the Th1, Th2, Th17/Tregs ratio.

Tregs expressing PPARγ can inhibit the inflammatory response, maintain autoimmune tolerance, and play an important role in the progression of AS, mechanisms that have recently received much attention. Activation of PPARγ recruits Tregs and inhibit the inflammatory reaction of the vascular wall and slows down the progression of AS (Castrillo and Tontonoz, 2004). Activated by free fatty acids and their metabolites, PPARγ can increase the quantity of Tregs (Pacella et al., 2018; Atif et al., 2020), to promote Tregs to secrete TGF-β and IL-10, and consequently play a role in inhibiting vascular inflammation and anti-AS (Feuerer et al., 2009; Cipolletta et al., 2011). In addition, Tregs are recruited to VAT inflammatory areas by chemokines or via recognition of tissue-specific antigens, to promote the expression of PPARγ at inflammatory sites (Cipolletta, 2014). PPARγ may increase the number of Tregs by the ectopic co-expression of Foxp3 and PPARγ (Hamaguchi and Sakaguchi, 2012). PPARγ can promote the transformation of macrophages to the anti-inflammatory M2 type by regulating the gene expression related to M2 type macrophages, and also secretes cytokines such as TGF-β and IL-10, to indirectly induce the expression of Tregs, and inhibit the inflammatory response and resulting anti-AS effect (Le et al., 2018; Lin et al., 2021). The mechanism of involving PPARγ agonist pioglitazone inhibiting the progression of AS in ApoE^-/-^ mice may be achieved by regulating the TGF-β/Smad signaling pathway (Fan and Watanabe, 2003; Lei et al., 2010), or by regulating Th1/Tregs (Ali et al., 2020) and Th17/Tregs (Tian et al., 2017) cell levels by activating PPARγ. (Figure 3)**.** PPARγ and Tregs interact with each other to inhibit the immune inflammatory response and jointly play an anti-AS role.

## Conclusion

It is well known that a variety of complex factors can promote the occurrence and development of AS. The pathological process of AS includes the formation and rupture of arterial subintimal plaques and secondary complex lesions, such as calcification, thrombosis, and intraplaque hemorrhage. In essence, AS is a chronic and progressive inflammatory disease, in which inflammation and immune regulation are responsible for the pathological process of AS. In recent years, inhibition of the inflammatory response to increase plaque stability, prevent plaque rupture, reduce secondary thrombosis, has achieved good results, and represents a research hotspot in the prevention and treatment of AS. A large amount of evidence shows that PPARγ and Tregs interact with each other during AS progression, play a role in regulating immune cytokines, suppress the inflammatory response, and exert anti-AS effects. The main mechanisms of synergistic anti-AS effects include 1) the activation of PPARγ in adipose tissue, which increases the number of Tregs by inducing the expression of Foxp3, and inhibits the proliferation of effector T cells; 2) PPARγ promotes the differentiation of Tregs by inducing the polarization of macrophages to the M2 phenotype; and 3) the combined effect of PPARγ and Foxp3 ectopic co-expression increases the quantity and enhances the function of Tregs. In consideration of the basic and clinical evidence supporting PPARγ and Treg inhibition of the immune inflammatory response, the delay in the progression of AS, and stabilization of AS plaques, with the objective of suppressing immune inflammation, anti-AS PPARγ agonists or Tregs preparations may be proposed as effective prevention and treatment of AS.
